# P–T-t conditions of successive deformation stages during exhumation of orogenically thickened crust; a case study from the Aar Massif, Central Alps, Switzerland

**DOI:** 10.1186/s00015-025-00484-9

**Published:** 2025-06-12

**Authors:** Edwin Gnos, Josef Mullis, Christian A. Bergemann, Thomas Pettke, Emmanuelle Ricchi, Axel. K. Schmitt

**Affiliations:** 1 Natural History Museum of Geneva, University of Geneva, Route de Malagnou 1, 1208 Geneva, Switzerland; 2 Earth and Environmental Sciences, Rue des Maraîchers 13, 1205 Geneva, Switzerland; 3 Institute of Mineralogy and Petrography, University of Basel, Basel, Switzerland; 4 Institute of Earth Sciences, Heidelberg University, Heidelberg, Germany; 5 Earth and Environmental Sciences, University of Geneva, Rue des Maraîchers 13, 1205 Geneva, Switzerland; 6 Institute of Geological Sciences, University of Bern, Baltzerstrasse 1+3, 3012 Bern, Switzerland; 7 John de Laeter Centre, Curtin University, Perth, Australia

## Abstract

Obtaining precise pressure–temperature-time constraints on the history of exhumation of orogenically thickened crust using rock-forming minerals of greenschist-facies rocks can be a challenging task. Rare examples exist where structurally distinct hydrothermal mineralisations have been used to pin-point specific stages during this evolution. This study combines hydrothermal fissure-quartz fluid and solid inclusion data with Ti-in-quartz thermometry, solute thermometry, and fissure monazite-(Ce) Th-Pb ion probe dating in order to establish a link between hydrothermal mineral crystallisation and major faulting events in the Grimsel Pass study area, central Aar Massif, Switzerland. Six fluid inclusion populations in quartz are distinguished in the older, steeply NNW-dipping fissure at the well-known Zinggenstock locality, four can be identified in quartz in younger, vertical fissures. All data together constrain formation and subsequent stepwise growth and evolution of the fissures to a P–T-t range of 450 °C/440 MPa and 300 °C/240 MPa between c. 15 and 7 Ma. In quartz zones containing rutile-whiskers in fluid inclusions, Ti-in-Qtz thermometry yields temperatures comparable to fluid inclusion solute thermometry. The combined data indicate that the oldest cleft quartz generation formed c.15 Ma ago during reverse faulting at 450 °C/440 MPa. A major change in the direction of the regional stress field linked with onset of dextral strike-slip movements along the Rhone-Simplon-Centovalli fault system then led to predominant dextral strike-slip faulting starting at c. 12–11 Ma, at P–T conditions between 375 °C/320 MPa and 330 °C/230 MPa. At Zinggenstock, the original cleft becomes overprinted by sinistral shear zones, and fluid advection at 330 °C/230 MPa. This CO_2_-bearing fluid led at the Zinggenstock location to the formation of white mica (muscovite-ferriphengite) at the expense of chlorite. At Oberaar, renewed dextral strike-slip reactivation occurred between c. 10 and at 7 Ma at conditions of 330 °C/230 MPa to 300 °C/240 MPa. Our data document variable stress regimes, locally associated with focused fluid flow, across an approximate depth interval of 16.3–8.5 km (~ 440 to 230 °C) during unroofing of the orogenically thickened crust. Hydrothermal mineral formation ages precisely constrain the chronology of successive deformation events, thus offering valuable constraints for unravelling the mechanisms of tectonically and buoyancy-driven exhumation of peripheral domains of the NW European Alps. Together, these data permit to estimate exhumation and cooling rates independent of thermochronology.

## Introduction

Fissures (open veins with parallel walls) and clefts (large openings, commonly with curved walls) form during brittle tectonic activity and represent fluid-filled cracks where, due to chemical disequilibrium, interaction of fluid with the surrounding wall rock led to dissolution-crystallization reactions and mineral crystallization in the fissure. Alpine fissures mainly formed under conditions below 450–500 °C and below 450–300 MPa (Mullis et al., 1994; Mullis, 1996; Fabre et al., 2002; Sharp et al., 2005). During their formation, fissures are usually oriented perpendicular to foliation and lineation (e.g., Gnos et al., 2015). Clefts may transform to veins if continued shearing causes rotation of the clefts into the plane of flattening, leading to the expulsion of the fluid. However, stress field changes over time may also lead to fissure intersection (e.g., Steck, 1968; Ricchi et al., 2019), sometimes resulting in complex multistage fissure systems (e.g. Ricchi et al., 2020). Fluid-filled fissures or clefts become chemically disequilibrated during deformation, inducing a continuous, cyclic or episodic growth or dissolution of minerals in fissures and clefts through direct cleft deformation, collapse of cleft wall sections and related exposure to fresh wall rock. Similarly, chemical disequilibrium due to temperature and pressure dependent changes in fluid saturation levels may play a role (e.g. Poty 1969; Mullis, 1975, 1976a, 1976b; Mullis et al., 1994; Mullis, 1996; Sharp et al., 2005). Due to this, fluid-rock interaction may cause leaching and partial mineral dissolution from the wall rock leading to porosity (e.g., Mullis, 1983; Mercolli et al., 1984; Mullis, 1995; Heijboer, 2006). Cleft minerals in greenschist and amphibolite-facies rocks are commonly zoned, and contain different sets of aqueous saline fluid inclusions with sometimes small amounts of CO_2_ (e.g. Poty et al., 1974; Frey et al., 1980; Mullis et al., 1994; Mullis, 1996). Early grown minerals may become overgrown by other minerals, protecting them from dissolution or reaction with the evolving cleft fluid. While interaction of wall rock leaching with cleft mineralization commonly shows evidence for a locally closed system (e.g. Sharp et al., 2005), open-system behavior has also been observed at times (e.g. Mullis, 1976a; Mullis et al., 1994; Mullis 2011). In a brittle environment, this may lead to a switch from a lithostatic to a temporary predominance of hydrostatic pressure (Mullis, 1983; Mullis et al., 1994). Due to all these processes, the mineral association found in fissures is distinct from that found in the host rock and, as a whole does not represent an equilibrium paragenesis.

Whereas neo- or recrystallized minerals in greenschist to sub-greenschist facies ductile to brittle shear zones and fault gouges are usually < 100 µm in size (e.g., Rolland et al., 2009; Pleuger et al., 2012), fluid-enhanced growth in fissures leads to mm- or cm-sized crystals.

Monazite, a rare earth element (REE) phosphate, is commonly used for Th-Pb or U–Pb dating. Clefts typically form below ~ 450–500 °C (e.g. Mullis et al., 1994; Mullis, 1996). Monazite-(Ce) (hereafter called monazite) grows in clefts in most cases at temperatures below ~ 350 ºC (e.g., Mullis, 1996; Berger et al., 2013; Gnos et al., 2015; Bergemann et al., 2017, 2018) and thus well below temperatures at which Pb diffusion in monazite is activated (e.g., Cherniak and Pyle, 2008; Gardés et al., 2006). Fissure monazite radiometric ages therefore always yield crystallization ages. This is at contrast to monazite in rocks, where fluid assisted reequilibration can occur (Bosse et al., 2009; Didier et al., 2013; Harlov and Hetherington, 2010; Harlov 2011; Tartèse et al., 2011; Williams et al., 2011). By dating individual crystal growth domains in cleft monazites in situ, monazite crystallization durations can be deduced in some cases (e.g. Gasquet et al., 2010; Janots et al., 2012; Berger et al., 2013; Gnos et al., 2015; Grand'Homme et al., 2016; Bergemann et al., 2017, 2018).

**Fig. 1:**
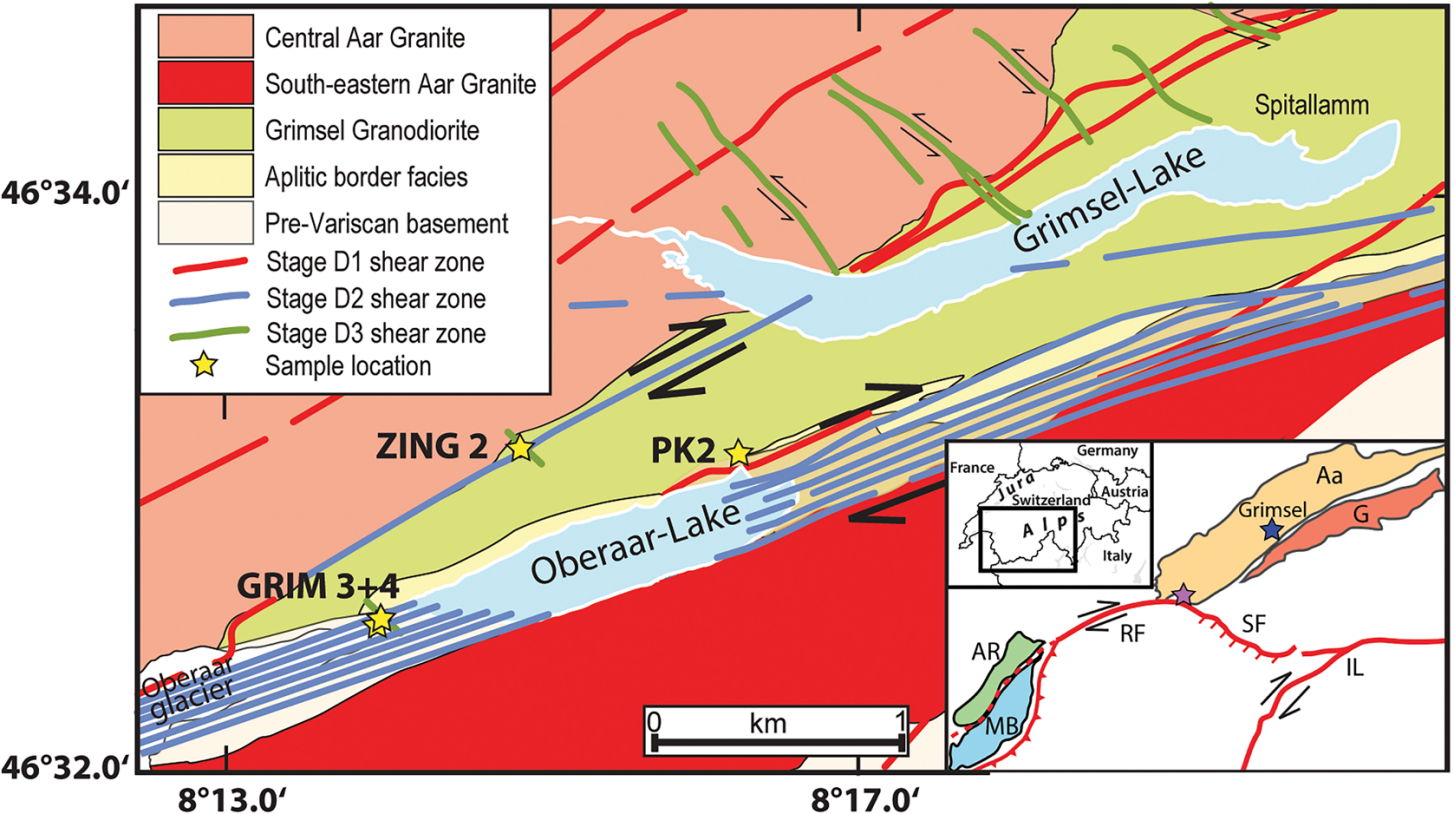
Geological map of the Grimsel pass region, Switzerland, based on Wehrens (2015). ZING2 (Zinggenstock), GRIM 3 + 4 (Oberaar) and PK2 (Oberaar-Lake) indicate localities of dated cleft monazites. Inset shows tectonic sketch with the locations of Aar Massif, Baltschieder Valley (light purple star), and the Simplon-Rhône line. Aa – Aar Massif, G – Gotthard Nappe, AR—Aiguilles Rouge Massif, MB—Mont Blanc Massif, RF – Rhone Fault, SF – Simplon Fault, IL—Insubric Line (after tectonic map of the Alps by Bousquet et al., 2012)

**Fig. 2:**
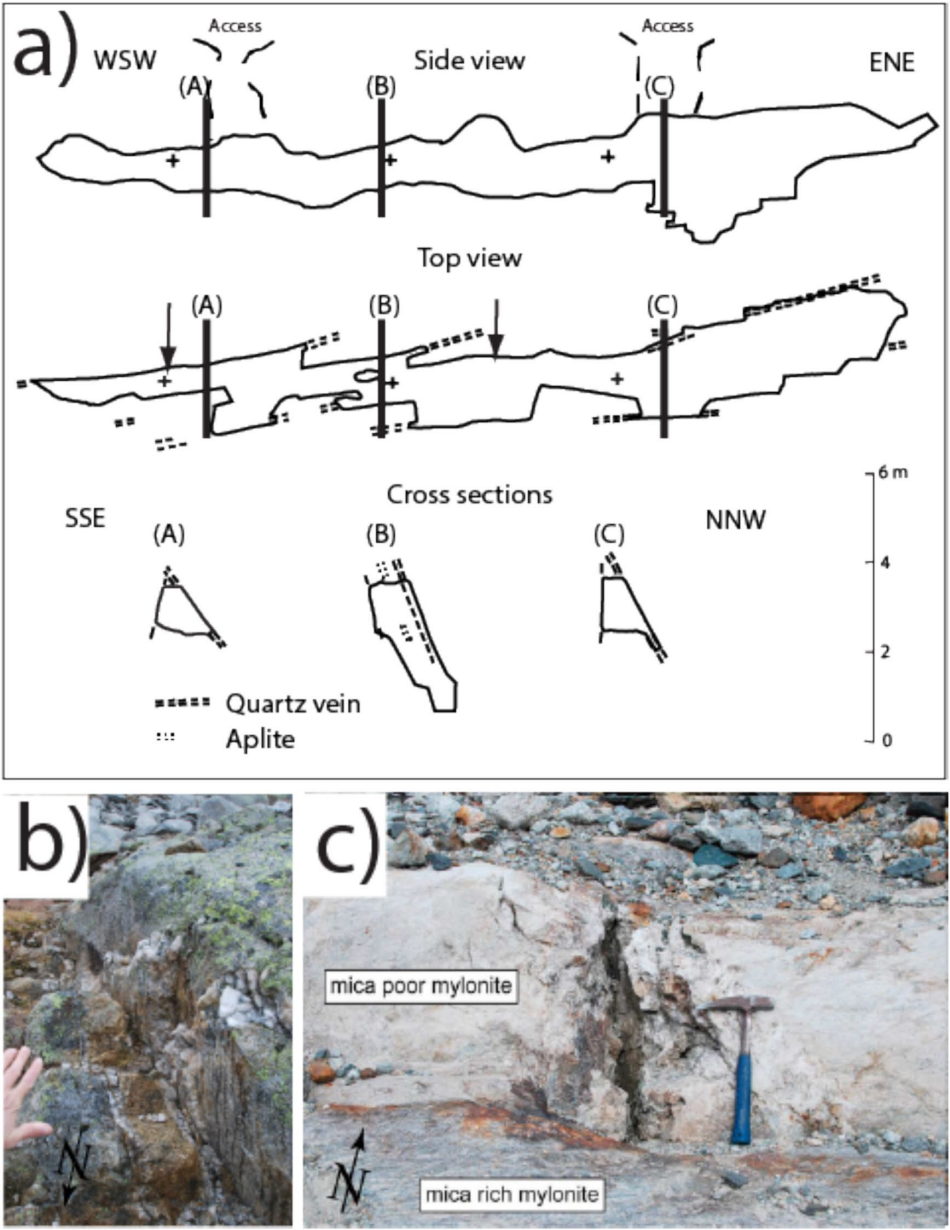
**A** Side view, top view and cross sections of the large fissure system in foliated Grimsel Granodiorite at Zinggenstock (site ZING 2 in Fig. 1) mapped by Josef Mullis after excavation. The fissures were accessed by two holes in the ground. The quartz veins mark the location of the four sub-parallel open fissures. **B** Late quartz-Mg-Fe-carbonate vein at the Zinggenstock site. **C** 40 cm large, vertical cleft (site GRIM 4 in Fig. 1), developed in the neck of boudins in more brittle deformed leucocratic mica-poor mylonite forming a band within more ductile deformed schistose, mica-rich mylonite

For the following reasons, the Grimselpass area located in the central Aar Massif, Switzerland (Fig. 1) represents an ideal location to study fissure evolution: (a) It is a classical, well-studied area for cleft mineral finds (Parker, 1954; Stalder, 1964; Stalder et al., 1998); (b) Much data exists on deformation and shear zone evolution (e.g. Marquer et al., 1985; Marquer, 1989; Challandes et al., 2008; Rolland et al., 2009; Goncalves et al., 2012; Wehrens 2017; Herwegh et al., 2017); (c) There exist many thermochronological data (e.g., Jäger et al., 1967; Purdy and Stalder, 1973; Dempster, 1986; Michalski and Soom, 1990; Challandes et al., 2008; Rolland et al., 2009; Rossi and Rolland, 2014; Rauchenstein-Martinek, 2014; Bergemann et al., 2017; Nibourel et al., 2021b); (d) Previous studies reported the occurrence of an older, horizontal, or at Zinggenstock NNW-dipping fissure system (e.g., Mullis, 1996) and a younger, vertical fissure generation (Bergemann et al., 2017), and (e) Mullis (1996) distinguished several quartz generations with the corresponding mineral parageneses and fluid inclusion populations in the Zinggenstock fissure system, trapped between 450 °C/440 MPa to 300 °C/240 MPa. Bergemann et al. (2017) determined fluid inclusion populations in quartz (Fig. 2) in a vertical fissure at the Oberaar glacier (location GRIM 3 + 4 in Fig. 1), trapped at 366 °C/316 MPa to 294 °C/252 MPa. These fluid inclusion data are compiled in Table 2.

Bergemann et al. (2017) also dated three cleft monazites from horizontal (location PK2 in Fig. 1) and vertical clefts (GRIM3 and GRIM4) located at the eastern and western ends of the Oberaar Lake in the Grimsel area, Switzerland (Fig. 1). Most of the Th-Pb monazite domain ages fell in a narrow range between 11.65 ± 0.40 Ma and 10.85 ± 0.36 Ma. One grain from a vertical cleft additionally yielded a younger growth domain age of 7.02 ± 0.31 Ma. They concluded that the main stages of monazite growth occurred coevally in horizontal and vertical clefts at c. 11.5 Ma, in connection with a major change in the regional stress field associated with “massif fluid flux” (Villa and Hanchar, 2013).

In this work we provide new solute thermometry for the Oberaar quartz fluid inclusions previously studied by Bergemann et al. (2017). We then compare these results with newly obtained Ti-in-quartz thermometry and combine these results with new monazite Th-Pb ion probe dating of two grains from the Zinggenstock fissure system (ZING 2 in Fig. 1) to constrain the pressure–temperature-time (P–T-t) evolution of hydrothermal cleft mineralization in the Grimsel area of the Aar Massif. Finally, we link these data with known changes in regional deformation regimes. This allows us to constrain the exhumation history of a crustal gneiss unit using cleft mineral crystallization ages and place its history in the context of progressive exhumation of peripheral tectonic units in the NW European Alps.

## Geological and tectonic setting

The Alpine orogen is the result of the closing of the Tethys Ocean, followed by collision of the Eurasian with the Apulian plate (e.g., Schmid et al., 2004). Shortening during orogenesis was accommodated by thrusting and lateral escape of rock units, resulting in the formation of shear zones. The Grimsel study area is situated in the southern part of the Aar Massif (Fig. 1), a 115 km long and 23 km large (N-S extent), paraautochthonous crystalline unit in the Central Swiss Alps deriving from the European margin. The massif consists of pre-Mesozoic polymetamorphic ortho- and paragneisses and migmatites, intruded by Variscan (e.g., c. 300 Ma old Grimsel Granodiorite) and post-Variscan magmas (e.g., Labhart, 1977; Abrecht, 1994; Schaltegger, 1994; Berger et al., 2016; 2017). Metamorphism during the Alpine orogeny reached greenschist facies peak conditions of 450 °C and 650 MPa (Challandes et al., 2008; Goncalves et al., 2012) before unroofing and cooling started at c. 19 Ma (Wehrens, 2015; Herwegh et al., 2017) which was associated with strong faulting.

At least four phases of deformation are reported for the Grimsel area in connection with the Alpine exhumation during which the crystal-bearing open fissures formed (Mullis 1996; Hofmann et al., 2004; Challandes et al., 2008; Rolland et al., 2009; Goncalves et al., 2012; Rossi and Rolland, 2014; Diamond and Tarantola, 2015; Wehrens, 2017). These deformation stages (D1 – D4) are:

Stage D1 ductile shear zones formed during NW directed thrusting in the biotite stability field (Handegg phase of Wehrens 2017). They strike NE-SW to ENE-WSW, show steep foliation and steeply SE plunging lineation, with mainly reverse faulting shear sense (e.g., Rolland et al., 2009; Wehrens, 2017). Where fluid was available and under lithostatic pressure, very little stress was necessary to provoke fluid-assisted fracturing (e.g., Mullis, 1995). Horizontal clefts formed during the evolving Handegg-phase in this stress field also outside the immediate vicinity of the shear zones.

Stage D2 shear zones (Oberaar_a_ phase of Wehrens 2017) are rich in white mica and chlorite. They are equally NE-SW and ENE-WSW oriented, displaying subhorizontal to oblique, east plunging stretching lineations. Shear sense indicators show dextral strike-slip movement, and vertical fissures formed in this stress field are largely restricted to shear zones and their immediate surroundings (Rolland et al., 2009; Bergemann et al., 2017; Wehrens 2017).

Stage D3 strike-slip deformation was likely less pronounced than the previous deformation phases and formed dextral (Oberaar_b_ of Wehrens 2017) as well as sinistral (Oberaar_c_ of Wehrens 2017) faults, showing chlorite and quartz ± Fe-bearing carbonate crystallization. Dextral D3 faults have an ENE-WSW orientation similar to stage D2 deformation. The WNW-ESE to NW–SE oriented sinistral D3 shear zone network, well developed in the Zinggenstock area (Rolland et al., 2009; Fig. 1, is described as conjugated with the dextral D3 strike-slip faults.

Stage D4 deformation led to a dextral brittle reactivation of ENE-WSW oriented shear zones with formation of cataclastic fault gauges (Kralik et al., 1992; Pleuger et al., 2012), and local hydrothermal brecciation at around 3.3 Ma (Hofmann et al., 2004; Belgrano et al., 2016).

## Cleft location and sampling

The cleft belonging to an older generation studied here is in foliated Grimsel Granodiorite at Zinggenstock (locality ZINGG 2 in Fig. 1; Mullis, 1996; 46º33.0’N/008º14.85’E; 2740 m). The second cleft studied belongs to a younger vertical generation and is in an aplitic rock in mylonitic, white mica and chlorite rich gneisses at the western end of Oberaar lake (locality GRIM 3 + 4 in Fig. 1; Bergemann et al., 2017; 46º32.31’N/008º13.87’E; 2240 m).

**Fig. 3:**
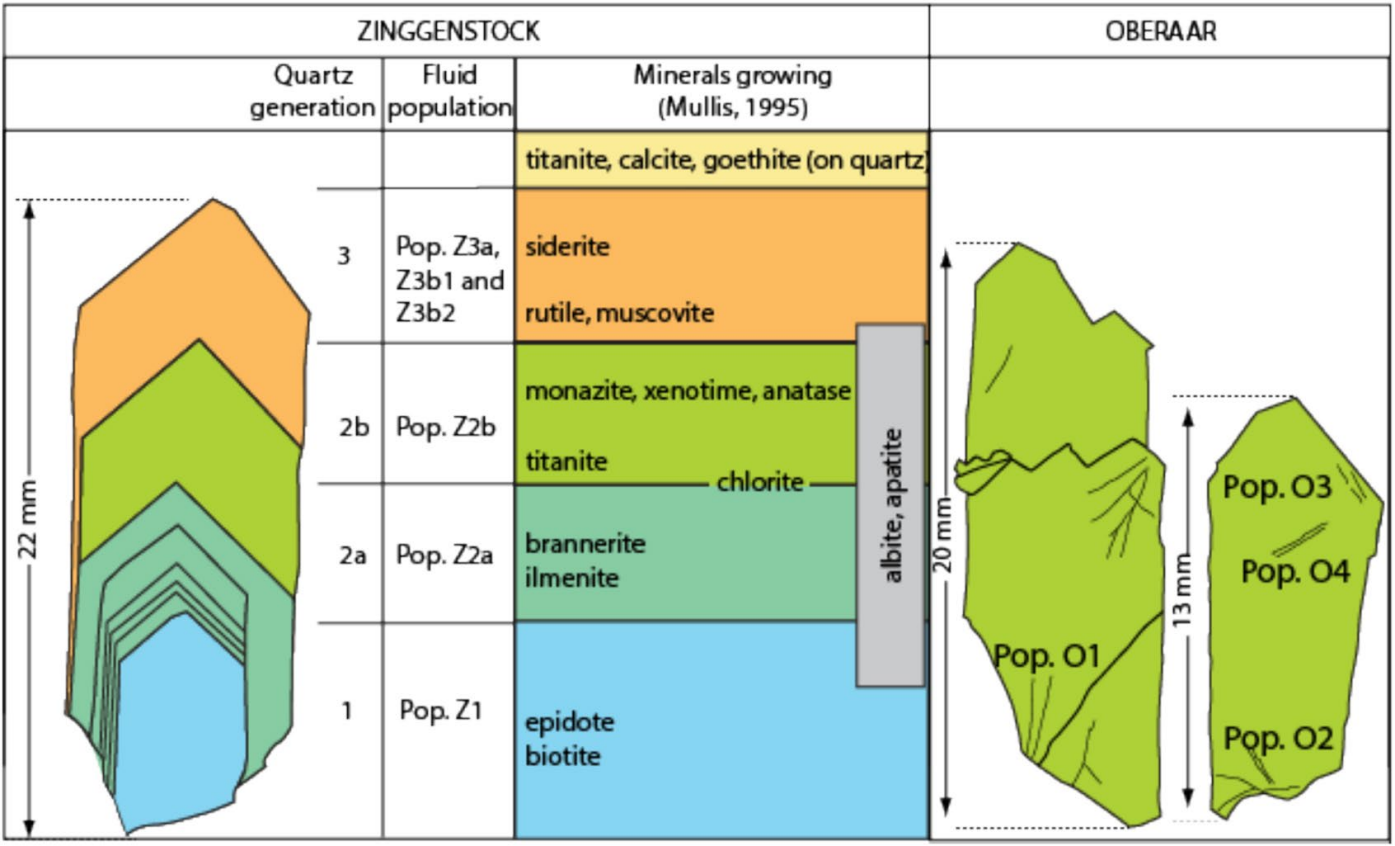
Drawing of investigated quartz thick sections from Zinggenstock (ENE striking and NNW dipping en-echelon fissure system) and Oberaar (later formed vertical fissure). The different colors indicate the main growth generations. At Zinggenstock, large quartz crystals containing abundant mineral inclusions (Mullis, 1995) indicate changing fluid composition. Note that at Zinggenstock monazite crystallizes at the end of quartz generation Z2b. Note that the Oberaar quartz crystallized mainly under conditions of generation 2b at Zinggenstock

The Zinggenstock cleft system (e.g., Mullis 1995, 1996; Fig. 2a) shows a complex shape consisting mainly of four subparallel, large clefts (striking 065–080° and dipping with 50–80° to NNE), connected by an irregular network of smaller fissures and quartz veins. The entire system has a total length of > 40 m and is underground. Although the mineral assemblage quartz, biotite and epidote are only found in the Grimsel area in clefts that formed during the D1 deformation stage, the present complex shape indicates tectonic overprinting of the initial fissure system by D3 sinistral shearing (to produce an en-echelon cleft system). The frequently changing crystallization conditions are reflected in different quartz generations and corresponding fluid and solid inclusion populations (Mullis, 1995; 1996; Fig. 3).

The Grimsel Granodiorite consist of primary plagioclase, quartz, K-feldspar, biotite, amphibole, titanite and apatite and secondary clinochlore, epidote and muscovite/sericite. It does not contain monazite. The minerals in the Zinggenstock cleft comprise, according to Mullis (1983), quartz, clinochlore, albite, white mica (muscovite-ferriphengite), anatase, rutile, ilmenite, titanite, calcite, epidote, biotite, apatite, monazite-(Ce), xenotime-(Y), brannerite, siderite and goethite (Fig. 3).

The leucocratic mylonitic granite hosting the Oberaar fissure (Fig. 2) is in a stage D2 shear zone and consists mainly of K-feldspar, plagioclase, quartz. The surrounding mylonitic gneiss bands are rich in muscovite and chlorite. The fissure mineral association in Oberaar includes quartz, clinochlore, albite, ilmenite and monazite-(Ce) but the crystallization sequence cannot be deduced for all minerals from the material available. The quartz samples analyzed in this study are from the same material used for fluid inclusion studies (Mullis, 1996; Bergemann et al., 2017).

In the Zinggenstock cleft, monazite was reported as small inclusion in quartz, crystallizing at the end of growth stage 2b of Mullis (1983; 1995; 1996; Fig. 3). Unfortunately, this sample was not available for our study. However, monazite also crystallized in pores that formed by leaching quartz and biotite from the host rock surrounding the cleft. These pores had connection to the fluid-filled cleft room. To establish a link between P–T estimates obtained on fluid inclusions and the time of monazite crystallization in the Zinggenstock (Fig. 3) fissure system, we selected two grains from the ZING2 sample (= Natural History Museum of Geneva sample number MHNG 490.050) for ion microprobe Th-Pb dating. We selected a characteristic, well-zoned crystal from the Zinggenstock cleft for Ti-in-Qtz thermometry and the two previously studied quartz crystals from the Oberaar cleft (Grim 3 + 4 locality in Fig. 1) for fluid inclusion solute thermometry and Ti-in-Qtz thermometry.

## Analytical techniques

Raman spectroscopy of solid phases (rutile whiskers) in fluid inclusion was performed on a Bruker Senterra system, using an excitation laser wavelength of 532 nm at the University of Basel.

Laser ablation inductively coupled plasma mass spectrometry (LA-ICP-MS) measurements were conducted at the University of Bern using a Lambda Physik GeoLas pro 193 nm ArF Excimer laser system coupled with an Perkin Elmer ELAN-DRCe quadrupole ICP-MS. Measurements closely followed procedures detailed in Pettke et al. (2012), employing an energy density of 16 J cm^−2^ on the sample surface at 10 Hz laser repetition rate and variable spot diameters of 30, 40, or 60 μm, larger than the fluid inclusions to ensure complete and controlled ablation. 120 μm large spots were used for Ti-in-quartz analysis and placed within a growth zone or next to the analyzed fluid inclusion of fluid inclusion plains. The ICP-MS settings were optimized to maximum signal to background intensity ratios with (^232^Th^16^O)^+^ production rates tuned to below 0.2% and robust plasma conditions as monitored by equal sensitivities of U and Th. Data reduction was done using SILLS (Guillong et al., 2008) with improved calculation of the limit of detection (Pettke et al., 2012), by summing the major element oxide totals to 100% (e.g., Gray, 1985) for host quartz and employing microthermometrically determined NaCl equivalent concentrations for fluid inclusion quantification. Synthetic SRM 610 glass from the National Institute of Standards and Technology (NIST) was used as the external standard.

**Fig. 4:**
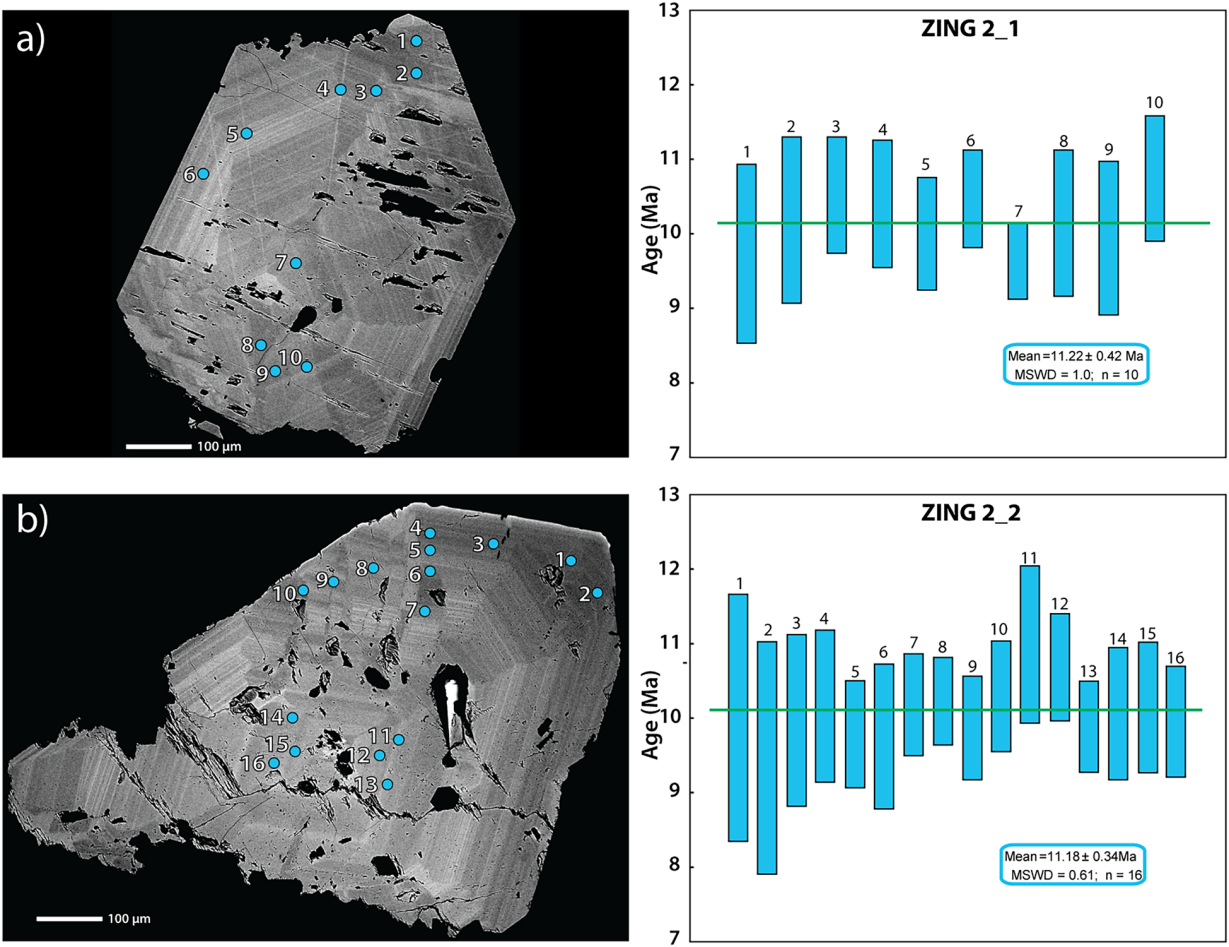
Monazite dating. For grains **a** ZING2_1 and **b** ZING2_2 on the left side BSE images with analysis spots marked as ovals to scale, on the right side Th-Pb spot ages and weighted mean ages. Scale bars are 0.1 mm

The separated monazites were polished down to the level of a central cross section, and backscatter electron (BSE) images were obtained on a JEOL JSM7001F electron microscope at the University of Geneva using a low beam current to avoid damage to the epoxy. Growth domains visible in BSE images generally correlate with variations in Th concentrations and were used to place ion probe (SIMS) spot analyses (Fig. 4) in order to capture the crystallization history. Measurement spots located near cracks or holes were avoided, as it was observed that the Th-Pb system could be disturbed in such areas, especially when later interaction with hydrothermal fluids is expected to have occurred (Janots et al., 2012; Berger et al., 2013; Bergemann et al., 2018).

Cathodoluminescence (CL) observations were performed on polished thin sections on a cold CL OPEA32J instrument equipped with a Leica DMLB microscope at the Natural History Museum of Geneva. The measurements were carried out at a 15 keV accelerating voltage and a 43–45 mA beam current. Digital images were taken with a ColorView IIIu camera controlled by the analySIS docu 5.0 software from Olympus Soft Imaging Solutions GmbH.

For dating of hydrothermal monazite, the U isotopic system is often not usable due to the unquantifiable incorporation of ^230^Th, a long-lived decay product of the ^238^U-^206^Pb decay series, into the monazite crystal lattice, which results in excess ^206^Pb (see detailed discussion in Janots et al., 2012). Moreover, Th concentrations are generally much higher than U in hydrothermal monazite (e.g., Grand’Homme et al., 2016). For these reasons the ^208^Pb/^232^Th system is used for dating the monazite domains.

Th-Pb analyses were carried out at Curtin University on a CAMECA IMS 1300HR3 SIMS instrument equipped with a Hyperion H201 radio frequency plasma ion source. Analytical methods followed Catlos et al. (2020), using high-energy ions of − 40 eV relative to the nominal accelerating voltage of + 10 kV and an energy bandpass of 50 eV. Secondary ions were generated by bombardment with a mass-filtered ^16^O^−^ primary beam of nominally ~ 5 nA focused into a 10 µm diameter spot. Counts were integrated after a 30 s–15 µm raster pre-sputtering and automated secondary beam centering in seven analysis cycles using a single electron multiplier at a mass resolution of ~ 5000 (M/ΔM) sufficient to resolve critical isobars. The exception was ^144^Nd^232^Th^16^O_2_^++^ on ^204^Pb which was unresolved under these conditions, and therefore the ^204^Pb intensities were corrected for stripping the interference relative to measured ^143^Nd^232^Th^16^O_2_^++^. The high-energy analysis approach minimizes monazite matrix effects, but yields approximately ten times lower abundances of high-energy secondary ions. Th-Pb data were calibrated against monazite reference 44,069 (424.9 ± 0.4, Aleinikoff et al., 2006), analyzed before and after the unknowns on two non-consecutive days. Lead isotope signals were corrected for common Pb contribution using measured ^204^Pb and an assumed present-day Pb isotope composition according to the model of Stacey and Kramers (1975). A correction factor for ^204^Pb accounting for the intensity difference with and without energy offset was determined by analyzing ^208^Pb under both conditions. Age calculations use the decay constants recommended by Steiger and Jäger (1977), and plots were done using Isoplot v. 3.71 (Ludwig, 2009). Th-Pb ages presented in the results section were corrected for common Pb and are given at 2σ uncertainties. IND and GM3 reference monazites were analyzed as secondary references and average ^208^Pb/^232^Th ages of 518.0 ± 6.6 Ma (mean square of weighted deviates MSWD = 0.071; n = 10) and 497.4 ± 5.5 Ma (MSWD = 0.033; n = 10) were obtained, which agree with reported ages of 509 Ma and 486.5 Ma within 1.7 and 2.2%, respectively (Lu et al., 2020). This is despite significant differences in Th abundances relative to 44,069, which for GM3 are ~ 130% higher than for 44,069.

## Results

### Quartz trace element analysis

Trace element concentrations in the different quartz growth domains (Fig. 3) described by Mullis (1996) and Bergemann et al. (2017) were obtained by LA-ICP-MS and results are listed in Table 1. The Al, Ti, Na, and Li concentrations are typical of fissure quartz (e.g., Jourdan et al., 2009).

**Table 1:** Quartz trace element contents and Ti-in-Qtz thermometry

Locality	Domain	^7^Li	^23^Na	^27^Al	^49^Ti	^57^Fe	TiQ W&W	TiQ T2010	TiQ H&A
		µg/g	µg/g	µg/g	µg/g	µg/g	°C	°C	°C
Zinggenstock	Pop Z1	1.91	0.06	14.49	0.38	1.31			
Zinggenstock	Pop Z1	1.88	0.05	13.63	0.37	1.65			
Zinggenstock	Pop Z1	1.58	0.22	10.93	0.34	1.87			
Zinggenstock	Pop Z1	1.42	0.13	10.44	0.32	1.76			
Zinggenstock	Pop Z1	1.80	0.17	13.05	0.34	2.30			
Zinggenstock	Pop Z1	1.84	0.30	12.84	0.34	2.85			
Zinggenstock	Pop Z1	1.75	0.14	12.86	0.31	2.29	**(338 ± 27)**	(283 ± 22)	**(366 ± 29)**
Zinggenstock	Pop Z2a	2.49	0.10	18.55	0.46	2.28			
Zinggenstock	Pop Z2a	9.75	0.43	75.02	1.76	3.00			
Zinggenstock	Pop Z2a	7.51	0.43	56.36	1.44	1.60			
Zinggenstock	Pop Z2a	5.36	0.23	40.19	0.99	1.56			
Zinggenstock	Pop Z2a	7.01	0.30	52.53	1.21	2.15			
Zinggenstock	Pop Z2a	2.76	0.13	20.10	0.46	2.57			
Zinggenstock	Pop Z2a	4.50	0.09	33.73	0.64	2.06	**388 ± 194**	305 ± 154	**394 ± 200**
Zinggenstock	Pop Z2b	5.32	0.08	39.93	0.70	1.90			
Zinggenstock	Pop Z2b	2.52	0.22	18.16	0.54	2.49			
Zinggenstock	Pop Z2b	1.76	0.46	13.37	0.36	1.89			
Zinggenstock	Pop Z2b	1.64	0.05	12.23	0.32	1.94			
Zinggenstock	Pop Z2b	1.40	0.11	10.14	0.26	2.10	**(353 ± 143)**	(276 ± 114)	**(355 ± 147)**
Zinggenstock	Pop Z3a	14.27	0.22	109.73	0.42	1.96			
Zinggenstock	Pop Z3a	16.71	0.49	130.15	0.46	2.52			
Zinggenstock	Pop Z3a	15.20	0.53	115.89	0.41	2.61			
Zinggenstock	Pop Z3a	15.90	3.23	122.73	0.38	2.52			
Zinggenstock	Pop Z3a	16.98	1.41	129.54	0.40	3.44	**347 ± 24**	262 ± 18	**342 ± 24**
Zinggenstock	Pop Z3b1,2	7.34	0.38	58.21	0.30	3.00			
Zinggenstock	Pop Z3b1,2	11.04	1.10	79.69	0.29	4.50			
Zinggenstock	Pop Z3b1,2	7.84	0.40	58.03	0.37	3.46	**(336 ± 49)**	(259 ± 38)	**(337 ± 49)**
Oberaar	Pop O1	1.81	1.00	12.84	0.30	–			
Oberaar	Pop O1	1.90	0.20	13.39	0.28	–			
Oberaar	Pop O1	0.85	1.65	6.15	0.29	–			
Oberaar	Pop O1	1.66	1.33	11.59	0.28	–			
Oberaar	Pop O1	1.73	0.42	11.98	0.28	–			
Oberaar	Pop O1	1.90	0.33	13.10	0.29	–	**370 ± 23**	292 ± 18	**378 ± 24**
Oberaar	Pop O2	1.69	1.39	9.69	0.66	–			
Oberaar	Pop O2	1.26	0.49	7.64	0.64	–			
Oberaar	Pop O2	1.63	0.35	9.86	0.71	–			
Oberaar	Pop O2	1.62	0.31	8.88	0.73	–			
Oberaar	Pop O2	1.67	0.23	9.99	0.73	–			
Oberaar	Pop O2	1.68	0.14	9.99	0.65	–	**342 ± 22**	268 ± 17	**356 ± 23**
Oberaar	Pop O3	1.96	0.50	10.96	0.35	–			
Oberaar	Pop O3	0.99	0.43	5.36	0.39	–			
Oberaar	Pop O3	0.90	0.30	4.98	0.39	–			
Oberaar	Pop O3	0.87	0.04	4.81	0.35	–	**362 ± 24**	280 ± 18	**363 ± 24**
Oberaar	Pop O4	1.46	0.24	8.27	0.56	–			
Oberaar	Pop O4	2.39	0.24	13.76	0.60	–			
Oberaar	Pop O4	2.37	0.26	13.92	0.61	–			
Oberaar	Pop O4	2.34	0.16	13.98	0.61	–			
Oberaar	Pop O4	2.34	0.15	13.31	0.58	–			
Oberaar	Pop O4	2.14	0.03	12.85	0.53	–	**(331 ± 4)**	(250 ± 3)	(**326 ± 4)**

W&W: Wark and Watson (2006); T2010: Thomas et al. (2010); H&A: Huang and Audétat (2012). For the numbers in brackets, TiO_2_ activity is probably < 1 (fluid inclusion do not contain rutile whiskers). Temperature estimates in bold are comparable to fluid inclusion thermometry

**Fig. 5:**
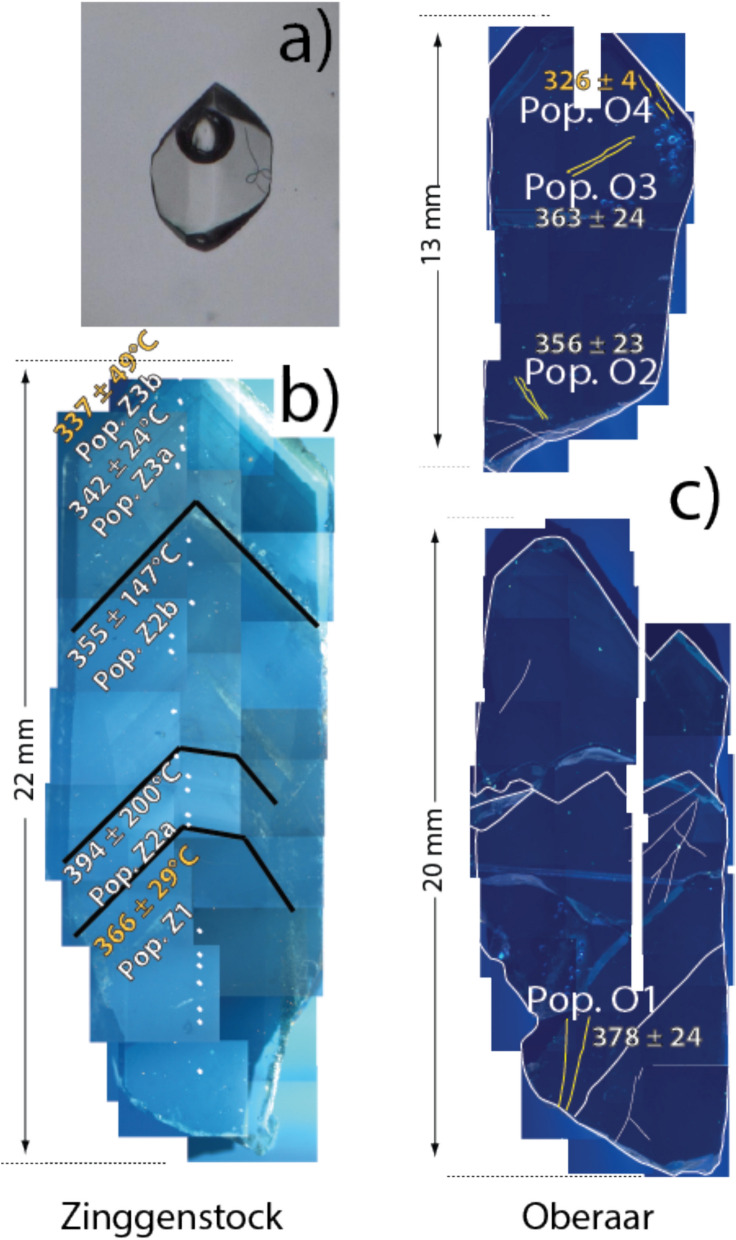
**A** Rutile whisker in a 0.106 mm long fluid inclusion from Zinggenstock used as indicator for Ti-saturation during the corresponding quartz growth stage. Photo Timon Kipfer. **B** Cathodoluminescene composite image of studied quartz from Zinggenstock. The growth stages are well recognizable and underlined by mineral inclusions. **C** Cathodoluminescence composite images of studied quartz crystals from Oberaar. The healed areas surrounding the secondary fluid inclusion populations O1-O4 show comparable dark blue luminescence as the original growth stage. Temperature indicated were obtained with the Huang and Audétat (2012) thermometer (Table 1). Orange numbers mark growth stages containing fluid inclusion without rutile whiskers

We measured trace element contents in the different quartz generations and healed zones containing primary, early secondary and secondary fluid inclusions (Fig. 5). At Zinggenstock quartz fluid populations Z2a to Z3a contain rutile whiskers (Fig. 5a), whereas at Oberaar, this is the case for quartz associated with fluid inclusion populations O1 to O3 (Table 2). The calculated Ti-in-quartz temperatures in growth zones containing rutile whiskers, using the Huang and Audétat (2012) calibration and the uncertainty from the Ti analyses, yielded temperatures of 394 ± 200 ºC and 342 ± 24 ºC for fluid populations Z2a and Z3a from Zinggenstock and between 378 ± 24 ºC and 356 ± 23 ºC for populations O1 to O3 from Oberaar (Table 1; Fig. 5). Ti-in-quartz temperatures in zones lacking rutile whiskers in the fluid inclusion population are listed in brackets. The Wark and Watson (2006) calibration yields similar results, whereas the Thomas et al. (2010) calibration yields much lower temperatures (Table 1) and pressure estimates systematically > 560 MPa.

**Table 2:** Summary of Zinggenstock and Oberaar quartz fluid inclusion data

Locality	FP	IT	nI	VoT	Vol% bubble	Daugh.M	TmIce °C	TdClathr °C	ThIncl °C	H_2_O mole%	NaCl mole%	CO_2_ mole%	T °C	P MPa	K^+^/Na^+^	T Giggenbach °C	T Can °C	NaCl wt% (1)	NaCl wt% (2)
Zinggenstock	Z1	psII	9	H_2_O	10 ± 1	Rt, A	-4.3 -4.2 / -4.4	-1.8 2.0 / 1.0	200 202 / 198 L	96.9	1.6	1.5	450	440					
Zinggenstock	Z2a	psII	27	H_2_O	9 ± 1	Rt, A, Phy	-5.1 -4.9 / -5.4	-3.7 6.5 / 3.0	213 216 / 207 L	96.1	2.1	1.8	385	330					
Zinggenstock	Z2b	psII	9	H_2_O	9 ± 1		-5.3 -5.0 / -5.4	-8.0 ± 0	228 230 / 234 L	95.4	2.1	2.5	375 ± 10	320					
Zinggenstock	Z3a	I	61	CO_2_	12 ± 1	Rt, A, Phy, black solid phase	-5.9 -5.3 / -6.3	-7.9 8.6 / 7.5	224 235 / 217 L	94.8	1.2	4.0	330 ± 10	230					
Zinggenstock	Z3b_1_	psII	55	H_2_O	6 ± 1	n.o	-5.1 -4.6 / -5.5	n.o	196 208 / 179 L	> = 96.4	2.6	< = 1	320*	260	–	–	–	–	–
Zinggenstock	Z3b_2_	psII	8	H_2_O	5 ± 1	n.o	-4.7 -4.6 / -4.8	n.o	139 143 / 130 L	> = 96.6	2.4	< = 1	300 *	240	–	–	–	–	–
Oberaar	O1	psII	20	H_2_O	9 ± 1	Rt, A	-6.0 -6.1 / -5.9	-6.0 5.0 / 7.0	206 199 / 209 L	95.5	2.5	2.0	366 ± 22*	316 ± 20	**0.374 ± 0.0029**	**366 ± 28**	**363 ± 28**	**9.21** **9.1/9.3**	**9.209**
Oberaar	O2	II	16	H_2_O	8.5 ± 1	Rt, A	-3.8 -3.8 / -3.7	5.7 5.0 / 6.5	197 190 / 201 L	96.6	1.4	2.0	344 ± 20*	294 ± 18	**0.287 ± 0.0035**	**338 ± 41**	**325 ± 40**	**6.14** **6.0/6.14**	**6.156**
Oberaar	O3	II	14	H_2_O	8 ± 1	Rt, A	-3.6 -3.7 / -3.5	4.1 3.5 / 5.0	187 183 / 191 L	97.1	1.2	1.7	325 ± 19*	279 ± 18	**0.207 ± 0.033**	**295 ± 48**	**276 ± 45**	**5.85** **5.7/6.0**	**5.861**
Oberaar	O4	II	15	H_2_O	6 ± 1	n.o	-1.7 -1.8 / -1.5	n.o	162 150 / 167 L	99.0	1.0	~ 0	294 ± 18*	252 ± 17				**2.89** **2.6/3.0**	**2.900**

New, complementary data are marked in bold. I = primary; II = secondary; psII = pseudo-secondary*; T, P = temperature and pressure obtained using a geothermal gradient of 30°Ckm^−1^; FP = fluid inclusion population; nI = number of inclusions measured; VoT = volatile type in fluid inclusions; n.o. = not observed; b.d. = below detection limit: Rt = rutile: A = anatase; Phy = phyllosilicate; L = homogenization into liquid phase. Giggenbach = Giggenbach (1988); Can = Can (2002); NaCl estimates based on fluid inclusion thermometry (1) and on LA-ICP-MS analysis of fluid inclusions (2). Most Zinggenstock data are from Mullis (1996)

### Fluid inclusion composition and solute thermometry

To complement results of Bergemann et al. (2017), the four different fluid inclusion populations observed at Oberaar (Fig. 6 in Bergemann et al., 2017) were analyzed by LA-ICP-MS in order to apply solute thermometry (Tables 2 and 3). The earliest fluid inclusion population is of early secondary, all others are of secondary nature (Table 2; Fig. 3). K and Na concentrations are continuously decreasing in fluid inclusion populations 1–3, whereas the As concentrations are highest in populations 2 and 3, reaching 1050 ± 360 µg/g. Whereas Li, B, Al, Rb, Sr and Ba were measured in all four fluid inclusion populations at concentrations below 400 µg/g, K, Ca, Mn, Fe, Pb were below detection in some of the populations. Th and U were detected only in the first fluid inclusion population at concentrations below 1 ppm (Table 3).

**Fig. 6:**
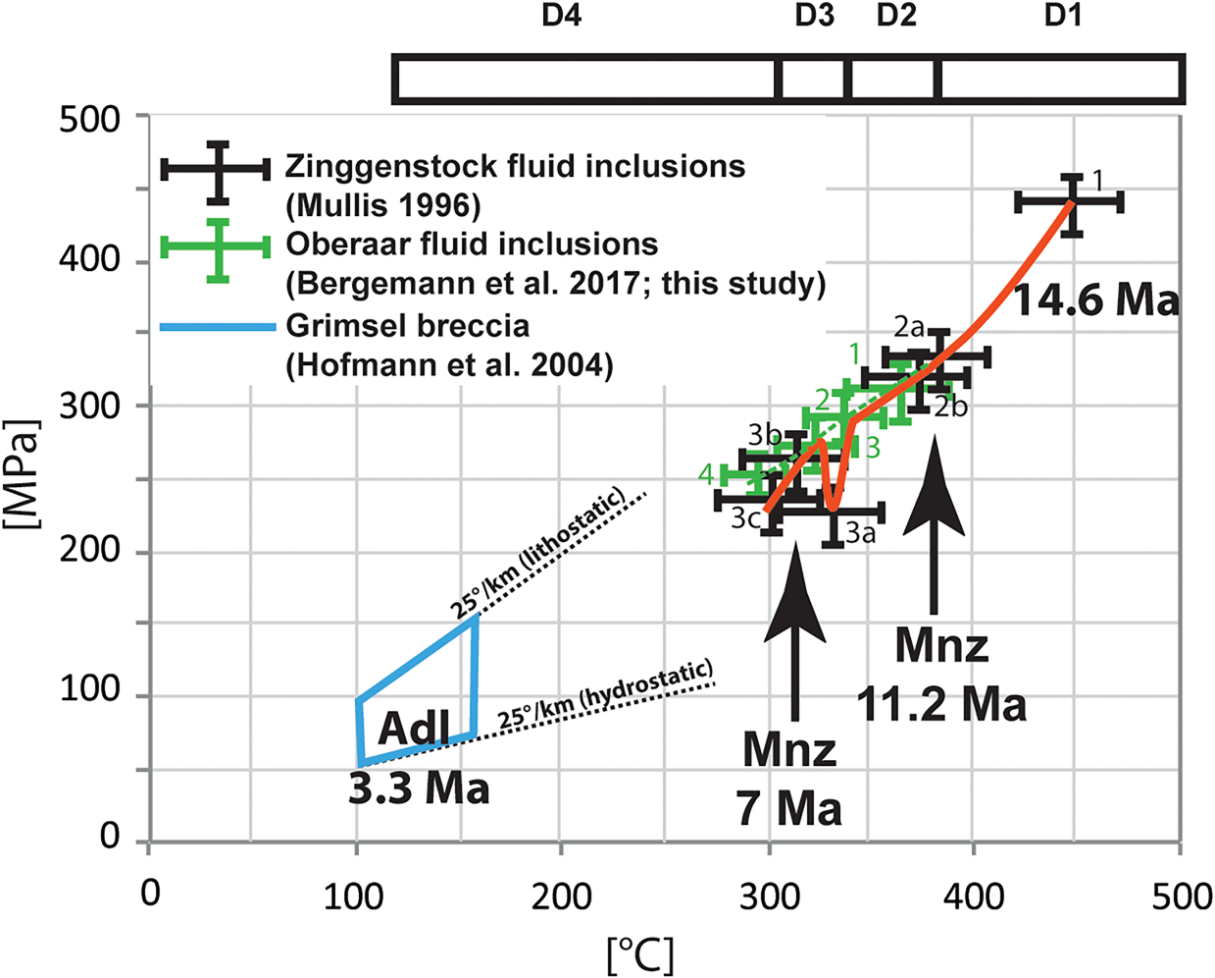
Compilation of fluid inclusion results, combined with monazite crystallization ages and deformation stages for the analyzed Zinggenstock and Oberaar quartz crystals. Based on biotite and epidote inclusions in cleft quartz at Zinggenstock and Spitallamm (Fig. 1), we assume that at both sites trapping of fluid inclusions during the D1 stage occurred at 14.6 Ma (Arnold, 2022). D4 (brittle reactivation of D2 dextral strike-slip faults) predates the crystallization of hydrothermal adularia (Adl) dated with ^40^Ar-^39^Ar at 3.30 ± 0.06 Ma (Hofmann et al., 2013) but could be as old as the latest fluid generation in the Oberaar cleft showing already dilution by meteoric water. Note the pressure drop recorded in fluid population 3a in the Zinggenstock cleft (Mullis, 1996; dashed red line). This small pressure drop is only recorded in the fluid inclusion population 3a at Zinggenstock (Mullis, 1996)

**Table 3:** Oberaar quartz fluid inclusion composition

Population		^7^Li	^11^B	^23^Na	^27^Al	^35^Cl	^39^ K	^43^Ca	^55^Mn	^57^Fe	^75^As	^85^Rb	^88^Sr	^137^Ba	^208^Pb	^232^Th	^238^U
		µg/g	µg/g	µg/g	µg/g	µg/g	µg/g	µg/g	µg/g	µg/g	µg/g	µg/g	µg/g	µg/g	µg/g	µg/g	µg/g
Pop. O1	average	250	290	32,900	68	63,700	12,380	2900	78	55	73	62	89	90	37	0.011	0.020
	stdev	25	36	770	46	11,200	900	670	11	26	13	10	8	20	10	0.012	0.016
Pop. O2	average	160	360	20,700	220	24,000	6000	b.d	6	106	1051	45	41	7	2	b.d	b.d
	stdev	34	40	430	100	4600	700	b.d	5	64	350	11	13	3	1	b.d	b.d
Pop. O3	average	140	310	20,600	95	25,800	4100	1020	b.d	b.d	560	30	76	17	b.d	b.d	b.d
	stdev	38	30	400	55	4900	670	670	b.d	b.d	88	4	13	5	b.d	b.d	b.d
Pop. O4	average	100	210	11,380	65	23,200	b.d	b.d	b.d	180	9	23	10	8	b.d	b.d	b.d
	stdev	30	35	40	16	6100	b.d	b.d	b.d	195	7	3	1	1	b.d	b.d	b.d

b.d. = below detection limit

Applying the K/Na solute thermometers (Giggenbach, 1988) and propagating uncertainties from LA-ICP-MS Na and K analysis yielded temperatures of 366 ± 28 ºC, 338 ± 41 ºC and 295 ± 48 ºC for the earlier three fluid inclusion populations at the Oberaar cleft, in good agreement with fluid inclusion thermometry (Table 2) obtained by intersection of the isochores with the geothermal gradient of 30 °C/km (Bergemann et al., 2017). The Can (2002) equation gives similar estimates, though within error a somewhat lower temperature of 276 ± 45 ºC for population 3 (Table 2). Due to K concentrations in population 4 below detection limit (Table 2), solute thermometry could not be applied.

### ^232^Th-^208^Pb monazite geochronology

The radiogenic ^208^Pb of monazite analysis spots varies between 85.9 and 100%. Despite some zoning recognizable on the BSE images, the Th-Pb spot ages obtained in the different zone of the grain overlap (Fig. 4; Table 4). Measurement spots on the two crystals of sample ZING2 (Fig. 4) yield for grain ZING2_1 a weighted mean Th-Pb crystallization age of 11.22 ± 0.42 Ma (MSWD = 1.0; n = 10) and for grain ZING2_2 of 11.18 ± 0.34 Ma (MSWD = 0.61; n = 16), identical within error.

**Table 4:** Ion microprobe Th-Pb age data

Analysis file name	^208^Pb/ ^232^Th age (Ma)	^208^Pb/ ^232^Th 1σ (Ma)	Radiogenic ^208^Pb (%)	^208^Pb/^232^Th	^208^Pb/^232^Th 1σ	^208^Pb (0 eV)/^208^Pb (-40 eV)	^143^Nd^232^Th^16^O2^++^/^204^Pb	^232^Th/^238^U	^232^Th/Th	^232^Th/Th 1σ
Grain 1										
Zing2_1@01.ais	10.55	1.00	92.4	5.22E−04	4.93E−05	12.17	0.14	36.2	0.94	0.02
Zing2_1@02.ais	11.30	0.93	95.9	5.59E−04	4.62E−05	12.44	b.d	22.7	0.90	0.02
Zing2_1@03.ais	11.86	0.65	94.9	5.87E−04	3.24E−05	13.85	0.21	59.0	0.82	0.01
Zing2_1@04.ais	11.66	0.71	91.3	5.77E−04	3.52E−05	12.56	0.12	38.6	0.84	0.02
Zing2_1@05.ais	11.00	0.63	97.5	5.44E−04	3.14E−05	13.4	0.33	58.5	0.87	0.02
Zing2_1@06.ais	11.77	0.54	98.6	5.83E−04	2.65E−05	11.93	0.42	48.6	0.88	0.02
Zing2_1@07.ais	10.37	0.42	91.5	5.13E−04	2.10E−05	13.29	0.14	59.2	0.80	0.02
Zing2_1@08.ais	11.24	0.82	85.9	5.56E−04	4.06E−05	13.01	0.15	37.5	0.86	0.02
Zing2_1@09.ais	10.90	0.86	94.0	5.39E−04	4.25E−05	12.29	0.24	38.2	0.88	0.02
Zing2_1@10.ais	12.23	0.70	96.3	6.05E−04	3.45E−05	13.52	0.35	44.1	0.79	0.02
Zing2_1@02.ais	11.30	0.93	95.9	5.59E−04	4.62E−05	12.44	b.d	22.7	0.90	0.02
Zing2_1@03.ais	11.86	0.65	94.9	5.87E−04	3.24E−05	13.85	0.21	59.0	0.82	0.01
Grain 2										
Zing_2_2@01.ais	11.00	1.38	100.0	5.44E−04	6.82E−05	13.12	1.47	67.2	0.6279	0.01579
Zing_2_2@02.ais	10.11	1.30	85.4	5.00E−04	6.44E−05	11.89	b.d	87.7	0.6465	0.01057
Zing_2_2@03.ais	10.95	0.96	94.3	5.42E−04	4.77E−05	11.94	b.d	93.3	0.6781	0.0149
Zing_2_2@04.ais	11.27	0.85	92.8	5.58E−04	4.20E−05	12.95	0.07	88.0	0.6426	0.01532
Zing_2_2@05.ais	10.64	0.60	96.1	5.27E−04	2.96E−05	13.02	0.23	124.1	0.6639	0.01263
Zing_2_2@06.ais	10.59	0.81	86.1	5.24E−04	4.01E−05	10.82	0.06	117.6	0.6673	0.01443
Zing_2_2@07.ais	11.30	0.57	92.8	5.59E−04	2.82E−05	10.65	0.14	215.5	0.6397	0.01298
Zing_2_2@08.ais	11.38	0.49	97.9	5.63E−04	2.42E−05	9.702	0.34	96.4	0.696	0.01071
Zing_2_2@09.ais	10.78	0.58	94.9	5.33E−04	2.86E−05	9.201	0.16	111.0	0.6989	0.01293
Zing_2_2@10.ais	11.49	0.62	98.7	5.69E−04	3.05E−05	10.13	0.40	100.6	0.6533	0.01462
Zing_2_2@11.ais	12.65	0.88	95.5	6.26E−04	4.36E−05	10.5	0.33	195.8	0.6258	0.01077
Zing_2_2@12.ais	12.14	0.60	97.5	6.01E−04	2.99E−05	10.3	0.39	217.4	0.6486	0.009776
Zing_2_2@13.ais	10.81	0.51	95.0	5.35E−04	2.50E−05	12.53	0.22	125.5	0.6007	0.009873
Zing_2_2@14.ais	11.10	0.74	93.1	5.49E−04	3.66E−05	10.76	0.18	207.0	0.5958	0.01164
Zing_2_2@15.ais	11.24	0.73	90.7	5.57E−04	3.60E−05	12.2	0.05	132.4	0.5791	0.007783
Zing_2_2@16.ais	10.92	0.62	96.7	5.41E−04	3.06E−05	11.72	0.27	160.0	0.6319	0.012
Zing_2_2@01.ais	11.00	1.38	100.0	5.44E−04	6.82E−05	13.12	1.47	67.2	0.6279	0.01579

All intensities measured at -40 eV energy offset except ^143^Nd^232^Th^16^O_2_^++^ and ^204^Pb common. ^208^Pb/^204^Pb = 38.63. All ages relative to monazite reference 44,069 (424.9 Ma). Grain 1 Pb/Th calibration slope = 0.3143; intercept = 0.0080 ± 0.008 (n = 20). Grain 2 Pb/Th calibration slope = 0.3116; intercept = -0.0198 ± 0.007 (n = 30). b.d. = below detection limit

## Discussion

### Cleft shape

Fissures occurring in boudin necks at Oberaar are vertical in orientation and of simple shape (Bergemann et al., 2017). This indicates that the stress field during D2 fissure formation and during D3 and D4 overprinting stages were of similar orientation. The complex geometry of the Zinggenstock cleft system, together with abundant fluid inclusion populations and solid inclusions in quartz, however, indicate a change in the orientation of the stress during formation and evolution stages. We interpret this cleft system as the result of deformation stages D1 (steeply south dipping reverse fault activity during the buoyancy-driven extrusion of the Aar Massif (Herwegh et al., 2017)) to D3. The switch from D1 reverse to D2 strike-slip faulting has been established at Oberaar to c. 12–11 Ma (Bergemann et al., 2017). Strike-slip deformation caused the formation of vertical quartz-Fe–Mg-carbonate veins at Zinggenstock (Fig. 2). The sinistral en-echelon cleft system shape (Fig. 6) at Zinggestock was probably caused by D3 deformation.

### Trace elements in quartz

The low Al, Na and Li content (Table 1) of the first main growth stages of quartz at Zinggenstock (Fig. 3) indicates macromosaic (Friedlaender) growth (e.g., Friedlaender, 1951), whereas the higher Li, Na, and Al concentrations in the last growth stage connected with fluid inclusion populations Z3a, Z3b_1_, Z3b_2_ (Table 2; Mullis, 1996) indicate growth of lamellar (Bambauer or Dauphiné) quartz (Bambauer et al., 1961) and thus faster quartz growth (e.g., Ramseyer and Mullis, 1990). Ti concentration show strong variation within some of the quartz growth zones at Zinggenstock (Table 1) resulting in large uncertainties. Fast lammelar growth is not observed in the studied quartz with macromosaic growth at Oberaar. It is known that trace element incorporation on different quartz growth faces is slightly different (e.g., Jourdan et al., 2009) and there is also experimental evidence that the Ti uptake shows variations according to growth rates (e.g., Huang and Audétat, 2012). However, the measured Ti concentrations are compatible with the temperature range obtained using solute thermometry.

### Thermometry

By comparing temperatures estimates obtained on quartz fluid inclusion populations from Zinggenstock and Oberaar, a correspondence between the four fluid inclusion populations found at Oberaar (Bergemann et al., 2017) to Zinggenstock Z2b, Z3a, Z3b_1_ and Z3b_2_ (Mullis, 1996) can be established (Table 2). This indicates that regional deformation events led to fluid inclusion trapping within both clefts.

In many cases, it is difficult to prove Ti-saturation during quartz growth. Here we measured Ti concentrations in all quartz domains associated with the different fluid inclusion populations. Rutile whiskers presence in many of the fluid populations (Fig. 5) is used as indicator that conditions of Ti saturation or close to Ti saturation were fulfilled, allowing a certain comparison between temperatures obtained by Ti-in-Q thermometry and fluid inclusion thermometry. The Huang and Audétat (2012) and Wark and Watson (2006) Ti-in-Q thermometers yield results consistent within error with fluid inclusion thermometry for the cases, where the presence of rutile whiskers in fluid inclusions (Fig. 5) indicate TiO_2_ activity of close to one (Tables 1 and 2). This is the case for fluid inclusion populations Z2b and Z3a at Zinggenstock (Table 2). However, in the first fluid population, the absence of rutile implies that the 366 ± 29ºC estimate obtained with the Huang and Audétat (2012) equation (assuming a TiO_2_ activity = 1) represents only a minimum temperature. It could be that biotite crystallizing during this stage (Fig. 3) incorporated the available Ti in its structure. In the Oberaar case, rutile whiskers occur in fluid populations 1–3 (Table 2; Fig. 5a) and the results of the Ti-in-Q thermometry and fluid inclusion thermometry again overlap. For stage 4 (lacking rutile whiskers), Ti-in-Q thermometry yields slightly higher estimates than fluid thermometry (Tables 1 and 2). The Thomas et al. (2010) Ti-in-Q thermobarometer calibrated for higher pressures and temperatures does not yield results consistent with estimates based on fluid inclusion data. Temperature estimates are much lower (Table 1) and pressure estimates are systematically > 560 MPa.

Based on hydrothermal experiments, Huang and Audétat (2012) suggested not to use the Ti-in-Q thermobarometer for hydrothermal quartz because growth rates were found to be strongly variable, thus potentially affecting equilibrium Ti-incorporation in quartz (see also Shulaker et al., 2015; Acosta et al., 2020). We also observed strong variations in growth stages Z2a (0.46–1.76 µg/g Ti) and Z2b (0.26–0.70 µg/g Ti; Table 1) at Zinggenstock. Our data do not provide evidence that the Ti incorporation was lower in the faster grown lamellar growth texture (Zinggenstock quartz generation 3 in Fig. 3), thus suggesting that quartz growth kinetics dependent Ti uptake did not occur for our samples (compare Huang and Audétat, 2012).

**Fig. 7:**
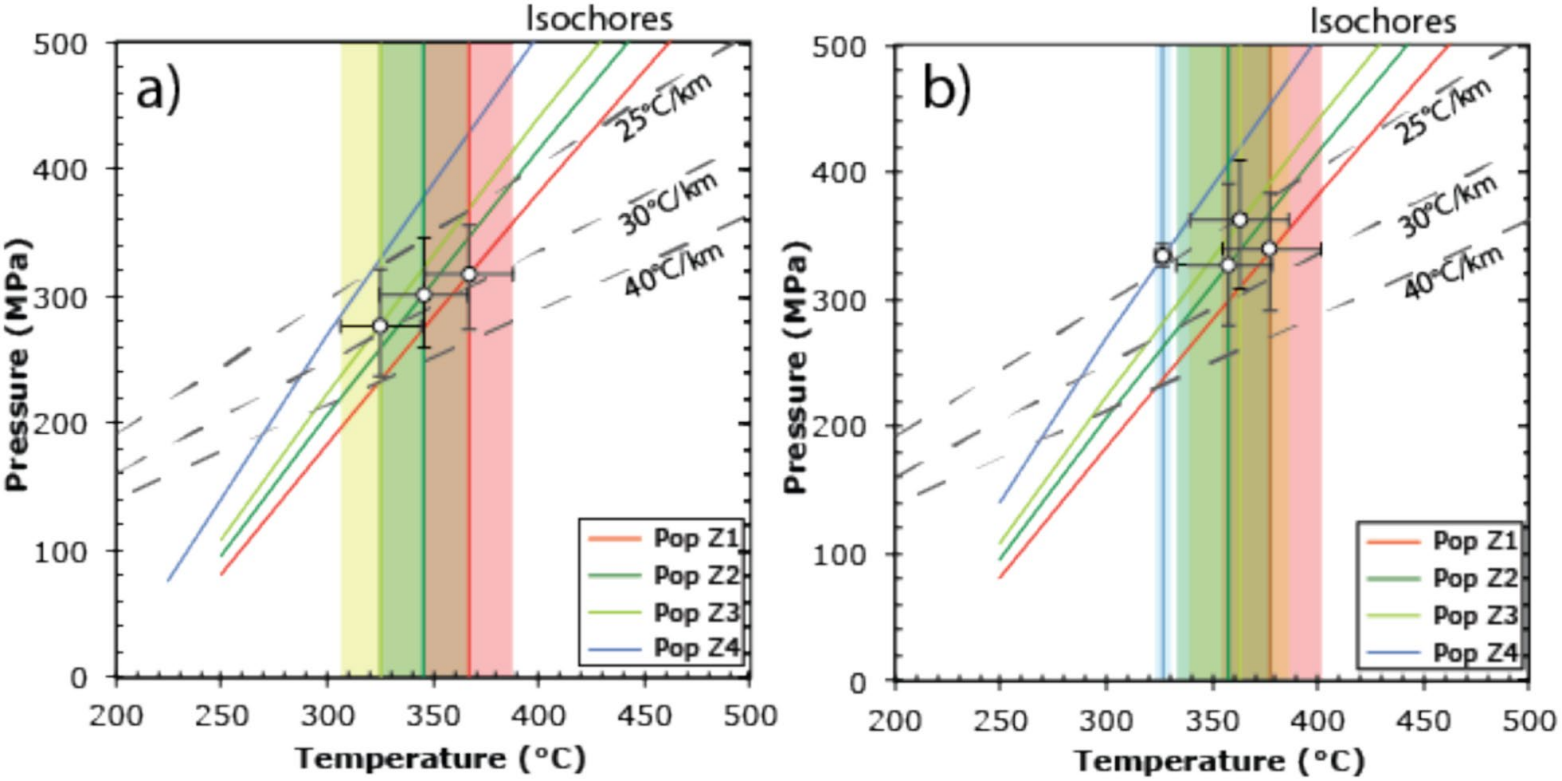
Lithostatic pressure–temperature diagram showing intersection points (white dots) of isochores of fluid inclusion populations from Oberaar with temperatures estimates obtained from fluid inclusion solute thermometry (**a**) and TitaniQ thermometry (**b**). Note that the lowest temperature obtained with TitaniQ thermometry is a minimum temperature (Table 1)

If we compare at Oberaar Na/K solute thermometry with temperature estimates obtained by intersection of fluid inclusion isochores with a lithostatic geothermal gradient of 30 °C km^−1^ (Bergemann et al., 2017), we see that both yield results overlapping within uncertainty (Table 2). Intersecting fluid inclusion isochores with temperatures derived from solute thermometry (Fig. 7a) or with temperatures obtained with the Huang and Audétat (2012) Ti-in-Q thermometer (Fig. 7b) also indicate that the thermal gradient was of the order of 30 °C/km.

### Cleft mineral crystallization ages

Monazite is usually not present in clefts in the Ca-enriched Grimsel Granodiorite (Fig. 1), but epidote-allanite and titanite are the REE-bearing minerals. Indeed, these minerals and brannerite crystallized in the early stages of quartz growth at Zinggenstock (Fig. 3). However, it is likely, that an aplite (Ca-poor but rich in U and Th) crosscutting the granodiorite wall rock at Zinggenstock may have played a major role in the system’s later evolution, possibly in connection with the advection of the CO_2_-bearing fluid. Titanite and epidote are not stable in a CO_2_-bearing fluid. It is presumed that leaching of this aplite (Fig. 3), probably in combination with the release of REEs, Th and U form titanite and epidote caused monazite to crystallize together with xenotime at the end of quartz generation 2b of Mullis (1996; Fig. 3). Since in both dated monazites all spot ages are overlapping within error (Fig. 4), indicating rapid crystallization, we assume that all monazites crystallized coevally.

If regional cooling age data are used for estimating the age of trapping of the earliest fluid inclusion population at 450 °C/440 MPa (Fig. 6), they indicate trapping at 19 ± 2 Ma (Mullis 1996). Comparable to the Zinggenstock occurrence (Fig. 3), biotite and epidote were also found as inclusions in quartz in a cleft in Grimsel Granodiorite at Spitallamm dam (Arnold, 2022; Fig. 1), yielding a biotite-epidote-adularia Rb–Sr isochrone of 14.62 ± 0.12 Ma. Since biotite was enclosed in quartz, protecting it from transformation to chlorite and from Rb–Sr equilibration with other minerals, the isochrone was interpreted to provide the time of biotite crystallization, and not a cooling age. If we assume that cleft biotite grew at Zinggenstock around the same time, this offers the possibility to base the age of entrapment of fluid inclusion populations (Fig. 3) on crystallization ages of fissure minerals only (fissure monazite Th-Pb data (Bergemann et al., 2017; this study) and biotite-epidote-adularia Rb–Sr isochrone (Arnold, 2022)). Therefore, the earliest fluid inclusion population of 450 °C/440 MPa associated with biotite and epidote would have been trapped around 14.62 ± 0.12 Ma (Table 5; Fig. 6). ^40^Ar-^39^Ar geochronology of hydrothermal cleft adularia in the Grimsel area yielded ages between 11.51 ± 0.42 Ma and c. 11.5–14.5 Ma (Rossi and Rolland, 2014; Rauchenstein-Martinek, 2015), which were interpreted as crystallization ages or cooling ages. Cleft monazite of 11.21 ± 0.22 Ma to 10.85 ± 0.36 Ma from horizontal and vertical clefts at Oberaar (Bergemann et al., 2017: Fig. 1) indicate that monazite crystallization in the entire Grimsel zone, affected by the change from reverse to strike-slip faulting, occurred mainly in this time interval. The two monazite Th-Pb ages of 11.22 ± 0.42 and 11.18 ± 0.34 Ma at Zinggenstock fall in the same age range. For this reason, we assume that monazite included in quartz at Zinggenstock also crystallized at that time.

**Table 5:** Correlation between fluid P–T estimates, mineral assemblages and cleft mineral crystallization ages

Locality	Fluid inclusion population	Characteristics	Mineral assemblage	Formation temperature (°C)	Formation pressure (MPa)	Age of formation (Ma)	Deformation event	References
Zinggenstock	Z1	H_2_O	Qtz, Bi, Ep, Ab, Ap	450 ± 15	440 ± 20	14.62 ± 0.12	D1	Mullis (1996); Arnold (2022)
Zinggenstock	Z2a	H_2_O	Qtz, Brn, Ilm, Ab, Ap	385 ± 15	330 ± 20			Mullis (1996)
Zinggenstock	Z2b	H_2_O	Qtz, Chl, Ab, Ap, Ttn, Mnz, Xnt, Ant	375 ± 10	320 ± 20	11.20 ± 0.54	D2	Mullis (1996); this study
Zinggenstock	Z3a	H_2_O with 4 mol. % CO_2_	Qtz, Ms, Rt, Sdr	330 ± 10	230 ± 20		D3	Mullis (1996)
Zinggenstock	Z3b_1_	H_2_O	Qtz, Ms, Rt, Sdr	320 ± 10	260 ± 20		D3	Mullis (1996)
Zinggenstock	Z3b_2_	H_2_O	Qtz, Ms, Rt, Sdr	300 ± 10	240 ± 20		D3	Mullis (1996)
Oberaar	O1	H_2_O	Qtz*	366 ± 20	316 ± 20	11.21 ± 0.22	D2	Bergemann et al. (2017), this study
Oberaar	O2	H_2_O	Qtz*	344 ± 20	294 ± 18		D2	Bergemann et al. (2017), this study
Oberaar	O3	H_2_O	Qtz*	325 ± 19	271 ± 18		D3	Bergemann et al. (2017), this study
Oberaar	O4	H_2_O	Qtz, Mnz*	294 ± 18	252 ± 18	7.02 ± 0.31	D3	Bergemann et al. (2017), this study

Qtz: quartz; Bi: biotite; Ep: epidote; Ab: albite; Ap: apatite; Brn: brannerite; Ilm: ilmenite; Ttn: titanite; Mnz: monazite; Xnt: xenotime; Ant: Anatase; Ms: muscovite; Rt: rutile; Sdr: siderite. *At Oberaar, mineral inclusions in quartz are lacking. The mineral assemblages are therefore not known

Later crystallization of the rim of a monazite from Oberaar, dated at 7.02 ± 0.31 Ma (Bergemann et al., 2017) indicates a younger event leading to monazite growth. At the Oberaar locality (Fig. 1), vertical, undeformed chlorite and chlorite-quartz veins surrounded by a bleached zone are common and probably formed during this event. Comparable undeformed veins (Fig. 2b) are also observed at the Zinggenstock site where they crosscut the complex cleft system, indicating a regional event, albeit without corresponding young ages having been measured. For this reason, we interpret the ~ 7 Ma domain ages obtained at Oberaar to be the result of monazite dissolution-crystallization during the entrapment of fluid population Z3a, which records a temporary pressure drop in the cleft (Fig. 6).

### Fluid record

The microthermometric study of Mullis (1996) revealed six fluid inclusion populations related to different quartz growth generations at the Zinggenstock cleft site (Fig. 3) trapped at conditions of 450 °C/440 MPa to 300 °C/240 MPa. Mineral inclusions in the quartz generations at Zinggenstock reflect biotite stability in association with fluid population Z1, chlorite stability in association with fluid populations Z2a and Z2b, and white mica (ferriphengite) stability in association with fluid population Z3a (Mullis, 1995). The largest volumes of quartz crystallization occurred at Zinggenstock during quartz generations 1, 2a and 2b (Fig. 3), and at Oberaar during first stage. At Zinggenstock, this is directly linked to creation of porosity in the wall rock due to dissolution of quartz and biotite and followed by monazite crystallization in these pores. Incursion of a CO_2_-enriched fluid at Zinggenstock at the begin of growth stage 3 (Fig. 3; Table 2) led to 4 vol% CO_2_ in the related fluid inclusion population (Mullis, 1996) and to sudden mineral instability, fluid-mineral-rock reactions, and crystallization of Fe–Mg carbonate refilling parts of the cleft wall pores. Incursion of a CO_2_-bearing fluid was not detected at Oberaar (Bergemann et al., 2017). On the other hand, the Oberaar cleft located in a dextral strike-slip fault shows dilution by meteoric water during the latest deformation stage (Bergemann et al., 2017).

The early pseudo-secondary fluid inclusion population in quartz from the vertical Oberaar cleft (Fig. 3; Table 2) permits to constrain cleft opening towards the end of the second fluid inclusion population documented at the Zinggenstock site (Mullis, 1995, 1996) and hence to stage Z2 deformation at conditions of ≤ 375 °C and ≤ 320 MPa. The quartz crystals of the vertical Oberaar cleft also contain three additional sets of secondary fluid inclusion populations (Fig. 3; Table 2) indicative of continuing activity along shear zones in this area at temperatures as low as 290 °C.

Fluid inclusion thermometry indicates that fluid populations Z2b, Z3a, Z3b_1_ and Z3b_2_ of Mullis (1996) at Zinggenstock can be correlated with fluid populations O1, O2, O3 and O4 of Bergemann et al. (2017) at Oberaar, indicating that tectonic events leading to fluid trapping were not restricted to a specific fault, but are of regional scale. However, temporary opening of the cleft led to locally different fluid compositions. For example, the small increase in CO_2_ at Zinggenstock in population Z3a (Table 2) derived from an external source (Mullis, 1996). Moreover, since monazite crystallization occurred towards the end of fluid population Z2b, it becomes obvious that deformation events are well recorded fissures located in the damage zone of master faults, in this case forming a km-sized zone. This shows that fluid-filled clefts provide a very sensitive system able to record both ductile and brittle regional deformation, down to temperatures where only K–Ar dating on illite from fault gauges have so far provided age constraints about faulting activity (e.g., Kralik et al., 1992: Pleuger et al., 2012).

Fourcade et al. (1989) have shown that the δ^18^O values are up to 2‰ higher in rocks in the mylonitic shear zones in comparison with the undeformed rocks of the Aar Massif, indicating mixing with meteoric water. The youngest fluid population O4 at Oberaar, a cleft located in a boudinaged aplite band within the shear zone center, consists of > 99% H_2_O (Table 2), indicating possibly dilution of the original metamorphic cleft fluid with meteoric water (Bergemann et al., 2017). However, the fluid population that was probably coevally trapped at Zinggenstock lacks any evidence for the incursion of surface water. Localized hydrothermal activity involving meteoric water continues in the study region until today (Waber et al., 2017; Diamond et al., 2018).

### Linking ages and deformation stages

Early K–Ar mica dating attempts to constrain the cooling history of the Grimsel area yielded an age range from 20 to 14 Ma, while Rb–Sr dating of biotite yielded ages of 15.5 to 13.1 Ma (Dempster, 1986). However, some of these ages were inconsistent with the general cooling pattern of the Aar Massif (Nibourel et al., 2021b). Little deformed country rock gave partially reset Rb–Sr pseudo-isochrons of 25.1 ± 0.7 to 15.9 ± 0.4 Ma (Challandes et al., 2008). In combination with structural mapping, biotite and white mica (phengites) separates from D1 shear zones (Fig. 1) were therefore dated using the Ar–Ar method and interpreted to date crystallization rather than cooling. Challandes et al. (2008) and Rolland et al. (2009) obtained biotite Ar–Ar ages of 21.2 ± 0.7 to 16.7 ± 0.7 Ma for these shear zones. These ages for D1 deformation are in line with regional considerations of thrusting in the Aar Massif and corresponding deformation in the Helvetic Nappes (Pfiffner, 2010).

For D2 fault zones, white mica gave Ar–Ar ages of 13.8 ± 0.1 to 12.2 ± 0.2 Ma (Rolland et al., 2009). The youngest of these ages overlap with Rb–Sr whole-rock-mica ages of mylonites of 12.2 ± 1.3 to 10.0 ± 0.3 Ma (Challandes et al., 2008), slightly younger than zircon fission track (ZFT) ages for rocks from the Grimselpass area of between 13.1 ± 0.7 and 12.4 ± 0.6 Ma (Michalski and Soom, 1990). Initial monazite crystallization occurred in older horizontal and younger vertical clefts, including the Zinggenstock monazites analyzed in this study, at the same time of ~ 11.2 Ma.

These coeval ages of different minerals and isotopic systems having a range of closure temperatures (Ar–Ar muscovite, whole-rock-mica Rb–Sr in mylonites and ZFT) strongly indicate that the Ar–Ar and Rb–Sr ages represent mineral formation ages rather than cooling ages (e.g. Villa and Hanchar, 2013; Diamond and Tarantola, 2015). As both analyzed monazites show coeval growth (Fig. 4) and monazite is reported at Zinggenstock as small inclusions in quartz at the end of growth stage Z2b (Mullis, 1995; Fig. 3), it is possible to link monazite crystallization with the quartz fluid inclusion record (Fig. 6). This indicates that the “massive fluid flux” postulated by Villa and Hanchar (2013) occurred at conditions of 385–375 °C and 320–330 MPa and was related to a switch in the stress field and inverse to dextral strike-slip faulting dated to c. 12–11 Ma by Bergemann et al. (2017). This change in the faulting direction from vertical to horizontal was not abrupt, but stepwise, suggested by locally preserved oblique, ~ 45°-dipping, stretching lineations. At a larger scale, this event is linked to underthrusting of the European crust during formation of the Jura belt at 12–10 Ma and coeval strike-slip deformational tectonic activity of the Rhone-Simplon line and the associated Western Alpine shear zone network at the time of 14–11 Ma (e.g. Campani et al., 2010; Fig. 1).

After ~ 12–11 Ma, strike slip deformation continued and led to the reactivation of some of the stage D2 shear zones and to renewed crystallization of monazite at Oberaar at ~ 7 Ma during stage D3 deformation. This indicates that stage D3 of Rolland et al. (2009), equivalent of Oberaar_b_/Oberaar_c_ of Wehrens et al. (2017) occurred at ~ 7 Ma and conditions of ~ 300 °C and < 280 MPa. Comparable K–Ar ages of 9.7 ± 0.4 and 7.0 ± 0.3 Ma (Kralik et al., 1992) and 11.7 ± 0.3 to 8.3 ± 1.1 Ma (Pleuger et al., 2012) were obtained by dating synkinematic illite in fault gouges. This indicates that the final monazite crystallization at Oberaar occurred at 300 °C and 240 MPa or slightly above.

Young cleft monazite was also found in structurally similar clefts from the southwestern Aar Massif with ^232^Th-^208^Pb crystallization ages of 8.03 ± 0.22 to 6.25 ± 0.60 Ma (Berger et al., 2013; Fig. 1), in the western Lepontine dome with ages of 8.41 ± 0.17 to 7.02 ± 0.23 Ma (Bergemann et al., 2020), in the Aiguilles Rouges and Mont Blanc Massifs with ages of 8.24 ± 0.38 to 6.71 ± 0.23 Ma (Bergemann et al., 2019) and in the Belledonne Massif with ages of 7.2 ± 0.4 to 5.4 ± 0.5 Ma (Gasquet et al., 2010; Grand’Homme et al., 2016). We postulate that the strike-slip faulting in the Grimsel area is kinematically linked to faulting in the western Alps along the Rhone-Simplon fault system (e.g., Haertel et al., 2013, Campani et al., 2010, 2014; Bergemann et al., 2020) and dextral strike-slip faulting in the Belledonne Massif (Ornon-Roselend fault; Gasquet et al., 2010; Grand’Homme et al., 2016).

Fluid inclusion P–T data used to correlate with cleft mineral crystallization ages and deformation stages are compiled in Table 5. The youngest age of 3.30 ± 0.06 Ma is recorded by adularia that crystallized in a hydrothermal/geothermal fault breccia (Hofmann et al., 2004) oriented parallel to the strike-slip faults and belongs the youngest stage D4 fault activity in the study area (Fig. 6).

Biotite Rb–Sr crystallization and monazite crystallization age data of cleft minerals indicate that trapping of the younger fluid inclusion populations Z2b, Z3a, Z3b_1_ and Z3b_2_ (Table 2) occurred a few million years later than the estimates based on mineral cooling data (Mullis, 1996).

**Fig. 8:**
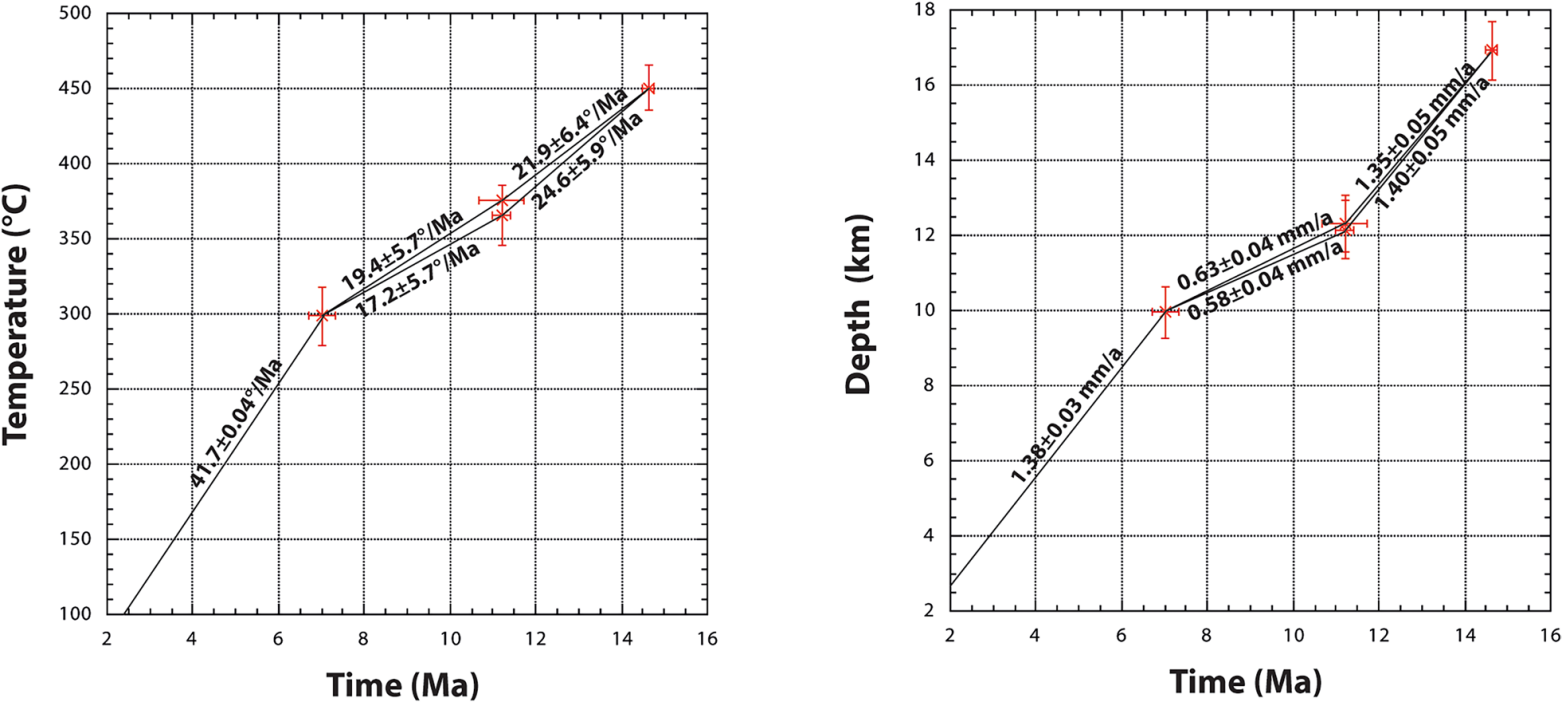
Average cooling (left) and exhumation rates (right) obtained by combining quartz fluid inclusion data with cleft mineral crystallization ages (Bergemann et al., 2017; Arnold, 2022; this study) and present average surface temperatures of 1.3 ± 5.7 °C at Oberaar, estimated from 1991–2020 data available on Meteoschweiz.admin.ch

The data in Table 5 can be used to estimate average cooling and exhumation rates, avoiding thermochronometry (Fig. 8). The cleft mineral crystallization data indicate an initial cooling rate of 22–25 °C/Ma, coupled with an exhumation rate of ~ 1.4 mm/a, followed by slower rates of 17–19 °C/Ma and 0.6 mm/a during the D2-D3 strike slip periods. This may indicate a slight down-movement of the study area during strike-slip activity and/or fluid-assisted heat advection, as presently observed in the Grimsel area (Waber et al., 2017; Diamond et al., 2018) and in other Alpine areas (Janots et al., 2019). From 7 Ma to present, the average exhumation rate is again 1.4 mm/a, associated with an increased cooling rate of 42 °C/Ma. This is likely due to strong glacial erosion during the last ~ 2 Ma and enhanced infiltration of meteoric water along the vertical faults. Present day uplift rates are about 1 mm/a (Schlatter, 2014).

This underlines the problem that in tectonically complex areas, where heat advection or meteoric water infiltration may occur along vertical fault zones, a distinction of crystallization and cooling ages may often be nearly impossible; hence, mineral crystallization ages are strongly preferred to constrain the timing of hydrothermal activity. However, Nibourel et al. (2021b) showed that when a large dataset of thermochronological data is available, local anomalies can be recognized and discarded. By doing this for the Eastern Aar Massif, they obtained exhumation rates for the Grimsel area comparable to our estimates.

## Conclusions

Following peak T-P conditions of 450 ± 25 °C and 650 ± 10 MPa at 22–17 Ma (Challandes et al., 2008; Rolland et al., 2009; Goncalves et al., 2012; Nibourel et al., 2021a), the current case study of fissures in the Grimsel area of the Aar Massif, Switzerland, documents the progressive exhumation of this crust across an interval of ~ 450—< 200 °C and at ~ 15—5 km depth (Mullis, 1975, 1976b; Bergemann et al., 2017) between ~ 15 and 7 Ma (based on crystallization ages). Changes in deformation styles from NW-directed thrusting in the beginning to E-W oriented normal faulting in response to lateral escape towards the end of this interval document the structural evolution related to unroofing of the Aar Massif. Comparisons with structurally similar tectonic units in the Alpine orogen (e.g., SW Aar Massif, Aiguilles Rouges Massif, Mont Blanc Massif, Belledonne and Pelvoux Massifs) show that late strike slip faults activity of comparable age affected all these peripheral domains of the exhuming NW Alps (e.g., Gnos et al., 2021). Our study indicates that in areas affected by vertical faults, where thermochonological data may be disturbed by fluid advection (Nibourel et al., 2021b), fluid inclusion P–T data linked with mineral crystallization ages provide an alternative to estimate cooling and exhumation rates independently. The study also shows that using in such tectonically complex areas an average paleo-thermal gradient for isochore-based pressure estimates can cause systematic errors.

Hydrothermal cleft mineralisations can offer a detailed record of the conditions prevailing during exhumation of orogenically thickened tectonic units, potentially covering a temperature window from ~ 500 to < 200 °C, at evolving pressure. Such data can be obtained from microthermometric fluid inclusion and mineral thermometry data. Fluid inclusion study may also reveal fluid of a different composition derived from a deeper source or from the surface mixing with the primary fluid, affecting the stability of previously crystallized minerals and lead to crystallization of new minerals. Because clefts represent fracture geometries that form in response to specific stress fields, their hydrothermal crystallization can be linked with the regional tectonic evolution of the crust. Late clefts typically form inside or near shear zones at abrupt rheological changes. Hydrothermal mineral formation ages then provide robust constraints on absolute timing of events. Together, hydrothermal clefts may offer unprecedented insights into the P–T-t evolution and tectonic processes of exhuming orogens, i.e., the retrograde metamorphic evolution that is very delicate to constrain using rock mineral data only.

## Data Availability

No datasets were generated or analysed during the current study.
